# Supplementary material for: Impact of network density on the efficiency of innovation networks: An agent-based simulation study
Source: PLoS One. 2022 Jun 17;17(6):e0270087. doi: 10.1371/journal.pone.0270087 (PMC9205519; doi:10.1371/journal.pone.0270087)
Supplement: Attachment — Submitted filename: Response to Reviewers.docx [file pone.0270087.s005.docx]

**Response to Reviewers**

We would like to thank the reviewers for the kind, highly academic and helpful comments. It is of great help to improve our research. In particular, some of the comments not only help us to refine this article, but also made us more confident of the value of this research. We also thank the work of the editor to help improve this study.

Now let us describe and reply to each comment one by one.

**Response to Reviewer #1:**

**Comment (1):**

More background literature should be added on the influence of network density on diffusion processes. For instance, network density influences the diffusion of disease processes on urban-like environments see: Nadini, M., Zino, L., Rizzo, A., & Porfiri, M. (2020). A multi-agent model to study epidemic spreading and vaccination strategies in an urban-like environment. Applied Network Science, 5(1), 1-30.

**Reply (1):**

We added more back ground literature on the influence of network density on diffusion process in the introduction **(line 37-line 46)**, and added three references (literature [11-13]) accordingly, including the one given by the reviewer.

**Comment (2):**

Figures 1-4 are all about different snapshots of the same ER with increasing values of p. These figures should, in my opinion, be moved on the Appendix or even removed because ER networks are very well studied. You can refer to the proper literature (see network percolation) to highlight the influence of the parameter p on a network

**Reply (2):**

We removed figures 1-4, and revised the corresponding narration **(line 226-line 229)**. And we presented the literature to find corresponding details on thresholds and phase transitions of Erdős and Rényi random network for interested readers.

We also added literature on percolation, and that helped to refine the review on network diffusion in the introduction **(line 37-line 46)**.

**Comment (3):**

The article does not study a realistic scenario and heterogeneity in degree distribution could be accounted. A way to easily generate arbitrary random networks is to follow: Perra, N., Gonçalves, B., Pastor-Satorras, R., & Vespignani, A. (2012). Activity driven modeling of time varying networks. Scientific reports, 2(1), 1-7. By aggregating several instances of a temporal network, a heterogeneous network is created. An even more popular approach is to use the configuration model to create random networks with heterogenous degree distribution. The Authors are free to explore and study different types of networks, which should encapsulate more realistic features

**Reply (3):**

We totally agree with the reviewer's suggestion, which will be very helpful to improve many of our studies. The given literature suggests a very good way to easily generate arbitrary random networks, and the configuration model could create random networks with heterogenous degree distribution, which could help us to encapsulate more realistic features.

In this paper, we focus on the important property of network density, in particular its impact on the system behavior in the context of innovation networks. In order to separate the contribution of network density from other network features (e.g., degree heterogeneity, clustering, etc.), the Erdős-Rényi model is considered to generate the test set of different structures.

As mentioned in extant literature, real networks are far more complex than classical models of random graphs, and most of such models are rather abstract and often unable to reproduce the structure of real-world networks in all aspects. Other parameters surely will have some influence on the experiments, and further the moderate the results of the model in this study. These suggestions point the way to further expand our findings in different areas in the future.

With the help of this comment, we refined the discussions and conclusions **(line 479-line 511)**, and the future research prospects **(line 578-line 580)**.

**Comment (4):**

Also, are results robust on variations of the size of the network N? For ER networks, there should be no problems at all in changing N, while for heterogeneous networks the parameter N may be an important factor.

**Reply (4):**

As the network density is defined as the ratio between the actual number of edges and the maximum possible number of edges in the network, and the maximum number of possible edges in a network of $N$ nodes is $C_{N}^{2}=N(N-1)/2$, so it is vital important to test the robustness of the results on $N$. We added more evidence (see figure 6) in the section of robust test. It further confirmed that the results are robust on variations of the size of the network.

As other features of the network, such as heterogeneity in some aspects (e.g., degree distribution, etc.) are not considered in this study. Yes, they may moderate the results in some extent, and that will be further explored in our future studies.

**Comment (5):**

Could the Authors please provide more information about the data availability? Would the Authors share a folder with codes?

**Reply (5):**

We added some more details of the model to make it better explained. Especially on the initialization of the knowledge vector and some settings of the interaction between nodes during the simulation process. We tried to make it more convenient for the readers to be able to replicate the process described in the text. We also supplement the data sets supporting the main findings of this study.

**Response to Reviewer #2:**

**Comment (1):**

The model needs to be better explained in two aspects.

First, when the authors say, at line 110, “the knowledge needed by the innovation is distributed in different nodes in the network”, it means that when initializing the vectors v_i the vector L is “divided” between the network nodes? This aspect needs to be better clarified also in subsection 4.1, “Parameters setting”, as the initialization of the network is not described.

Second, when the authors say, at line 122, “it is assumed that each node can communicate only once on a single topic at any given time”, does this mean that each couple of nodes, in each simulation, can communicate only on a specific topic? I think this mechanism is the crucial for understating the results the authors present.

Additionally, assuming to consider a node A and to select one of its neighbors B to interact, will B successively interact with another of its neighbors? In general, it might be extremely useful for the comprehension of the article if the authors furnished the code or at least a pseudo-code for their model.

**Reply (1):**

First, we added some explanation on how the knowledge of the innovation is distributed in different nodes at the start time: Here to model this idea, we randomly select $l$ nodes, set their knowledge vectors as {$k$, 0, …, 0}, {0, $k$, …, 0}, …, {0, 0, …, $k$} respectively. At the same time, set the knowledge vectors of other nodes all equal 0 **(line 112- line 114).**

Second, we added some explanation on how the nodes interacts in each time step (line 133-line 134). It means that each couple of nodes, at each time step (but not in each simulation experiment), can communicate only on a specific topic.

Additionally, we added explanation on how the nodes select their partners **(line 126-line 130).** Here if A selects B, then B will not be able to interact with others in this time step. If there is no idle node in the network, time t ends, and nest time step arrives, then both A and B will be able to select of be selected again.

By doing this, we made it more convenient for the readers to be able to replicate the process described in the text. We also supplement the data sets supporting the main findings of this study.

**Comment (2):**

Second, some aspects of the results need to be better clarified.

In Figs. 5 and 6, both the metrics NCTF and TTF have not been plotted for the entire interval of p. Does this mean that the cost and the time before innovation goes to infinity, i.e. the system never reach innovation? This would be coherent with the fact the PF is equal to 1, as this means that the failure of the innovation process is certain. However, by looking at Figs. 7 and 8, the cost and the time before innovations assume finite values even when PF=1. As these metrics, if I understood correctly, are finite only when the innovation occurs, how can their be so when PF=1? Such an aspect needs to be clarified.

Additionally, when the network is globally connected and given the model rules, the two metrics should be related by the relation NCTF = N*TTF(/ 2 , if each node interacts only once at each time step). Is this the case?

**Reply (2):**

First, the numbers of Figs 5 and 6 are changed to Figs 1 and 2 in the new version of manuscript. We added explanation on these tow figures **(line 257-line 261)**. The missing points of $NCTF$and $TTF$ mean infinity, and none innovation occurs during all the 10000 times of repeated experiments at this point of $p$ value.

Second, $PF$ in Figs 7 and 8 (Figs 3 and 4 in this version) are not equal to 1, but very near to 1. Every finite value of $NCTF$and $TTF$ in the figures corresponds to a $PF<1$.

Third, the relation between $NCTF$and $TTF$ is determined by the specific structure of the network. Assume the network is globally connected. If it is a complete network (one link exists between any pair of nodes), the two metrics should be related by the relation $NCTF=N*TTF/2$. Otherwise, it might be different. For example, a star with 1 core and $N-1$ non-adjacent peripheral nodes will have the relation of $NCTF=TTF$. Because in each time step, only 1 conversation could take place, and as the core node is occupied, most peripheral nodes will have to wait.

**Comment (3):**

Third, section 6, “Robustness tests”, needs to be improved. I would expect a figure or any other proof for each of the three aspects considered in the robustness analysis. Indeed, I think it is not correct to simply state that the experimental results show no significant changes without providing the outcome of these experiments.

**Reply (3):**

We added 4 figures as the proof of the three aspects of robustness tests. Also, we will upload all the date generated by these experiments.

**Comment (4):**

Fourth, in order to study the impact of density and to separate its contribution from the one of other network features (e.g. degree heterogeneity, clustering, …) , the authors consider the Erdös-Renyi model. However, such a model is rather abstract and it is often unable to reproduce the structure of real-world network. Can the authors point out to which extent the characteristics of the real-world innovation networks can be reproduce by such a model? I believe such an analysis can better help to assess the practical implications of their study.

**Reply (4):**

This comment is very helpful to refine the discussion and conclusions of this paper. We revised the corresponding part of **line 479-line 511**.

As mentioned in extant literature, real networks are far more complex than classical models of random graphs, and most of such models are rather abstract and often unable to reproduce the structure of real-world networks in all aspects. However, these models could capture some of the emerging properties not obvious at the level of their elementary constituents, and further provide the opportunities to investigate their striking consequences on the behavior of the system.

Our model focus on the property of network density and contributes to the network diffusion literature by inquiring the relationship between network density and two different diffusion regimes: exploration and exploitation. And we also contributes to the literature on Erdős-Rényi networks by analyzing how the branch formation in the Erdős-Rényi model impacts the diffusion process.

Specifically, this study contributes to the stream of literature on innovation networks by inquiring how network density affects the innovation efficiency of innovation networks. Extant research has shown equivocal evidences on this topic. This study analyzes the possible reasons behind the incoherent conclusions, and employs the multi-agent simulation method to disentangle this problem. The conclusions derived from the model results shed light on the theory and practice of innovation management, including the choice of innovation strategies of innovators and the formulation of innovation policies of innovation policy makers.
