# Supplementary material for: Impact of network density on the efficiency of innovation networks: An agent-based simulation study
Source: PLoS One. 2022 Jun 17;17(6):e0270087. doi: 10.1371/journal.pone.0270087 (PMC9205519; doi:10.1371/journal.pone.0270087)
Supplement: Attachment — Submitted filename: Response to Reviewers.docx [file pone.0270087.s006.docx]

**Response to Reviewers**

We would like to thank the editors and reviewers for their valuable comments and suggestions. We respond to every suggestion made by the editors and the reviewers in this revision. In particular, we attach great importance to the opinion of requiring a more in-depth analysis of the literature on agent-based models.

Now let us describe and reply to each comment one by one.

**Response to Reviewer #2:**

**Comment (1):**

On lines 456-457, the authors comment Fig. 7 stating that “The experimental results show that the change in l and k does not change the nature of the conclusions.” However, I think it would be worth further discussing the results displayed in such a figure. For instance, in the case of explorative innovation, the failure probability clearly depends on the size of knowledge vector l, while, for the exploitative innovation, this does not depend on the amount of necessary knowledge k. I think the authors should further comment on this outcome.

**Reply (1):**

We further discussed the results in Fig 7 **(lines 481-494)**.

Here we addressed the fact that “in the case of explorative innovation, the failure probability clearly depends on the size of knowledge vector l, while, for the exploitative innovation, this does not depend on the amount of necessary knowledge k”.

We also discussed the reasons of this outcome. And it is also shown that this result doesn’t change the conclusions extracted from the base case model, which are mainly about the relationships between the density of the network (p) and the parameters of innovation efficiency (TTF and NCTF).

**Comment (2):**

When discussing the robustness of their multiagent model to a variation in the “knowledge flow mechanisms in communications”, on lines 465-483, the authors simply claim that the nature of the conclusion remains unchanged. However, by inspecting Figs. 1, 8, and 9, one can notice some differences in the values of both the NCTF and the TTF, for both explorative and exploitative innovation. I think this result should be discussed in a revised version of the manuscript.

**Reply (2):**

We gave further discussions on this point **(lines 507-516)**.

Here we addressed this issue, explained the reason, and reconfirmed robustness of the results of the base case model.

**Comment (3):**

While it is extremely useful that the authors have provided the data generated from their numerical analysis, I believe that the multiagent simulation code should be made as well available. Indeed, since the model characteristics are central to the manuscript, I retain the availability of the code to be crucial.

**Reply (3):**

We upload the program file of our base case model to the submission website for review.

**Response to Reviewer #3:**

**Comment:**

The article deals with the questions to what extent network density regulates the diffusion of innovation. As such, the research question is relevant and pertinent. However, as already mentioned by the reviewers of the first round, I first recommend conducting a deeper analysis of the existing literature in this field with a particular focus on agent-based models, such as Mueller, M., Bogner, K., Buchmann, T. et al. The effect of structural is parities on knowledge diffusion in networks: an agent-based simulation model. J Econ Interact Coord 12, 613–634 (2017).

**Reply:**

We gave a deeper analysis of the existing literature in this field with a particular focus on agent-based models (**lines 26-59**). We also refined some narrative in this section, and added some references accordingly, including the one given by the reviewer.
